# The Effect of Four Weeks Dietary Intervention with 8-Hour Time-Restricted Eating on Body Composition and Cardiometabolic Risk Factors in Young Adults

**DOI:** 10.3390/nu13072164

**Published:** 2021-06-24

**Authors:** Su-Jeong Park, Jae-Won Yang, Yoon-Ju Song

**Affiliations:** 1 Department of Food Science & Nutrition, The Catholic University of Korea, Gyeonggi 14662, Korea; yh04264@gmail.com; 2 Department of Psychology, The Catholic University of Korea, Gyeonggi 14662, Korea; jwyang@catholic.ac.kr

**Keywords:** time-restricted eating, body weight, cardiometabolic risk factors, meal frequency

## Abstract

Recently, intermittent fasting, also known as time-restricted eating (TRE), has become a popular diet trend. Compared to animal studies, there have been few studies and inconclusive findings investigating the effects of TRE in humans. In this study, we examined the effects of 8 h TRE on body weight and cardiometabolic risk factors in young adults who were mainly active at night. A total of 33 young adults completed the 8 h TRE for 4 weeks. Body composition was measured by bioelectrical impedance analysis at baseline and every 2 weeks, and blood samples were collected at baseline and week 4. Daily dietary records were logged throughout the intervention period. Participants experienced significant changes in body weight (−1.0 ± 1.4 kg), body mass index (−0.4 ± 0.5 kg/m^2^), and body fat (−0.4 ± 1.9%) after 4 weeks of TRE. When participants were divided into weight loss/gain groups based on their weight change in week 4, fat mass reduction was significantly higher in the weight loss group than in the weight gain group. Regarding cardiometabolic risk factors, levels of fasting insulin and insulin resistance improved in the weight loss group after intervention, but not in the weight gain group. All subjects showed late-shifted sleeping patterns, but no significant differences in sleep duration, sleep quality, or psychological measures between the two groups. When meal frequency and energy proportion were evaluated, the average meal frequency was 2.8 ± 0.5 and energy proportions of breakfast, lunch, dinner, and snacks were 4.5, 39.2, 37.6, and 18.5%, respectively; there were no significant differences between the two groups. However, the saturated fat intake at dinner was lower in the weight loss group (3.1 ± 3.2%, 6.0 ± 2.5% respectively). In conclusion, 8 h TRE can be applied as a lifestyle strategy to manage body weight and cardiometabolic risk factors among young adults with late chronotypes.

## 1. Introduction

In recent years, intermittent fasting, also referred to as time-restricted eating (TRE), has become a popular diet trend as a simple weight loss method. TRE is a dietary strategy that cycles between eating and fasting periods. Organisms have evolved to limit their activities to certain times of the day, which influences their internal circadian clock and enables physiological processes to be performed at the optimal time [1]. Irregular meal timing can contribute to circadian rhythm disruption, which results in abnormal metabolic regulation and increased cardiometabolic risks [2].

TRE programs and their health outcomes have been well studied in animal models. Hatori et al. [3] found that mice subjected to 8 h time-restricted feeding and control mice exhibited similar weight changes, despite the time-restricted feeding group being fed isocaloric high-fat diets. Chaix et al. reported that TRE attenuated metabolic disease and body weight gain in mice subjected to various nutritional challenges such as high-fat diets and high-fat plus high-fructose diets [4]. Furthermore, transcriptomic and metabolomic analyses in circadian clock mutant mice further demonstrated the positive effects of TRE [5]. Together, the animal studies suggest that the beneficial effects of TRE occur via circadian rhythm and metabolic regulation.

Human TRE studies have largely been limited to observational studies [6,7]. Recently, several TRE intervention studies have been reported, although the findings are inconclusive. An 8 h TRE intervention study over 12 weeks in obese subjects showed significant body weight reduction in TRE subjects, but no differences in other metabolic risk factors such as serum lipids, fasting glucose, and fasting insulin compared to control or non-TRE groups [8,9]. Other TRE studies in subjects with metabolic syndrome or risk for type 2 diabetes showed improvements in metabolic parameters and glucose tolerance [10,11]. This suggests that the effects of TRE vary according to subject characteristics.

So far, human studies of TRE have focused on overweight or obese subjects, while few studies have been conducted on healthy young adults. A systematic review of 35 studies reported that young adults tend to have poor eating behaviors such as frequently consuming energy-dense snacks and eating at irregular mealtimes [12]. In particular, irregular mealtimes and late eating appear to increase cardiometabolic risk factors [13,14]. Meal regularity is often defined as three meals a day (breakfast, lunch, and dinner), and a study of Korean adults showed that subjects who consumed less than two meals per day had an increased risk of metabolic syndrome compared to those who ate three meals per day [15]. Young adults also tend to have irregular sleep patterns and high levels of sleep disturbance [16,17]. These sleep disorders are associated with cardiometabolic risk factors [18], and recent evidence suggests that meal timing is a modifiable risk factor for nocturnal awakening and sleep disturbance [19].

In this study, we examined the effects of 8-h TRE on body weight and cardiometabolic risk factors in healthy young adults and explored factors including meal patterns, sleep-related factors and psychological factors that could also affect weight.

## 2. Methods

### 2.1. Study Design and Participants

For this feasibility study, volunteers were recruited via flyers and online advertising between July 2020 and August 2020. Inclusion criteria were healthy young adults aged 18–28 who could engage in 4 weeks of TRE intervention. Exclusion criteria included previous diagnosis or treatment for metabolic diseases (e.g., diabetes, dyslipidemia, metabolic syndrome, etc.), having a weight change of 10% or more in the past month, or having a sleep disorder.

**Figure 1:**
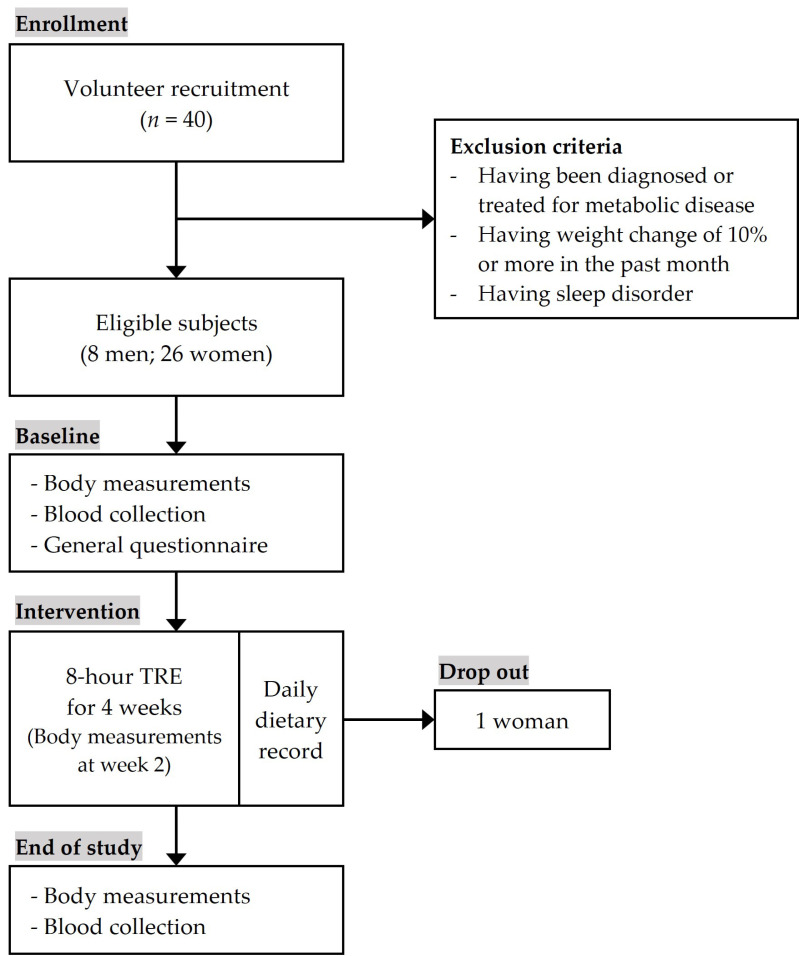
Study schematic diagram.

Of the 40 volunteers, 34 eligible participants were selected following screening by e-mail or phone call. After screening, participants were invited to attend a visit during which the purpose and procedures of the study were explained, and informed consent was obtained. At the first visit, a general questionnaire was administered, participants were instructed in how to complete dietary records and the first body composition measurements were taken. Within a few days, a blood sample was collected from each participant for biochemical measurements in a nearby hospital. Starting from the next day, all participants began 8 h TRE for 4 weeks. The participants were asked to select an eating window during their first visit, and for this to remain consistent throughout the intervention. There were no restrictions other than the time limit of the eating window. During the intervention, each participant reported their daily dietary record and sleep records via a mobile app. After 2 weeks, participants were invited to attend a second visit to measure body composition. After 4 weeks, participants made a final visit for body composition measurements and blood sample collection in a nearby hospital. The study scheme is presented in Figure 1.

This study was approved by the Institutional Review Board of the Catholic University of Korea (No. 1040395-202007-01) and registered using an online registration system as part of the Clinical Research Information Service (CRIS) (PRE20210-002) in South Korea, which is a primary registry of the World Health Organization International Clinical Trials Registry Platform (ICTRP).

### 2.2. Dietary Measurements and Meal Pattern Variables

Participants were asked to report their daily dietary records via a mobile app to track their dietary intake and evaluate meal patterns. All participants completed 28 days of dietary records. Participants were instructed to record descriptions of all foods and drinks consumed, the amount consumed, and the time of consumption. Participants were also asked to classify each meal as breakfast, lunch, dinner, or snack; the snack category was subdivided into light snack before breakfast, morning snack, afternoon snack, and late snack. Nutrient intakes were calculated from 28 days of dietary records using the Diet Evaluation System, a web-based program for the dietary assessment of Korean people [20].

To evaluate the meal patterns of participants, we analyzed the number of daily eating occasions, meal frequencies and meal proportions. Daily eating occasions were defined as the number of meals per day including breakfast, lunch, dinner, and four subcategories of snacks; a maximum of seven meals could be reported per day. Meal frequency was defined as the frequency of each meal type that was consumed per day over 28 days and meal proportion was defined as the average daily nutrient intake per meal over 28 days, in which four snacks were counted as a single meal. Nutrient intakes recorded for meal proportion included energy, carbohydrates, dietary sugar, protein, fat, and saturated fat.

### 2.3. Body Measurements and Body Composition Variables

Participants were invited for body composition measurements at baseline, after 2 weeks of TRE, and after 4 weeks of TRE. Body composition was measured via bioelectrical impedance analysis (InBody 230, Inbody Co., Seoul, South Korea) to obtain weight, muscle mass, fat mass, and percent of body fat. Height was measured using an extensometer (DS-102, DONG SAHN JENIX CO., TLD., Seoul, South Korea), and body mass index (BMI) was calculated using the equation BMI = kg/m^2^. Waist circumference was measured by a staff member using a measuring tape.

### 2.4. Biochemical Measurements and Cardiometabolic Risk Factors

Blood samples were collected at baseline and after 4 weeks for biochemical analysis. Whole blood was collected into serum-separating tubes (5 mL) and centrifuged to prepare the serum at the Catholic University of Korea Bucheon St. Mary’s Hospital. The samples were stored in a refrigerator and then analyzed by the Global Clinical Central Lab (GC Labs, Gyeonggi-do, South Korea). Insulin was analyzed using an electrochemiluminescence immunoassay, blood glucose was measured by ultraviolet spectrophotometry, and serum lipids such as total cholesterol, triglycerides, low-density lipoprotein (LDL)-cholesterol, and high-density lipoprotein (HDL)-cholesterol were measured by colorimetry (Cobas 8000, Roche., Basel, Switzerland). The cardiometabolic risk factors examined in this study included glucose metabolism and blood lipid parameters. For glucose metabolism, pre- and post-intervention fasting insulin and fasting glucose were compared. To evaluate insulin resistance, the homeostasis model assessment of insulin resistance (HOMA-IR) was calculated using the following formula: (fasting glucose × fasting insulin)/22.5 [21].

### 2.5. General Questionnaires and Other Variables

Participants were asked to complete a questionnaire at baseline, which consisted of questions about their basic characteristics, as well as lifestyle, sleep, and psychological variables. Basic characteristics included age, sex, and education level. Lifestyle variables included alcohol consumption, smoking, physical activity, and weight control. To evaluate alcohol consumption, we asked, “Have you ever had more than one drink in your life?” and, “How often do you drink?” From these questions, subjects were defined as “none” if they had never drunk alcohol, and “drinker” if they had drunk more than once per month in the past year. Smoking status was assessed by asking questions such as, “What is the total amount of cigarettes you have ever smoked?” and, “Do you smoke now?” Subjects were classified as “current smokers” when they answered, “More than five packs (100 pieces)” and, “Yes,” respectively; as “former smoker” when they answered, “Less than five packs (100 pieces),” and, “No,” respectively; otherwise, subjects were classified as “none”. Since the number of current smokers was very small, this variable was not considered in this study analysis. Physical activity was defined as “Yes” if participants performed high-intensity exercise for at least 75 min per week, or moderate-intensity exercise for at least 150 min per week, or a combination of high- and moderate-intensity exercise for at least 150 min per week.

Regarding sleep quality, subjects were asked questions such as, “How would you rate your overall sleep quality during the past month?” and, “How often have you had trouble sleeping because you cannot get to sleep within 30 min during the past month?” or, “How long (in minutes) has it taken you to fall asleep each night?” all of which were adapted from the Pittsburgh Sleep Quality Index, the most commonly used generic measure of sleep quality in clinical settings [22]. In addition, subjects were asked to indicate their self-perceived chronotype, i.e., morning/early type, evening/late type, or neither. Psychological measurements included 10 questions regarding self-esteem stability [23] and 21 questions regarding emotional responsivity, with the sub-factors: emotional sensitivity, emotional intensity, and emotional persistence [24].

### 2.6. Statistical Analysis

All continuous variables are presented as mean ± standard deviation and all categorical variables are presented as *n* and percentage (*n*, (%)). Differences in the basic characteristics of participants according to sex were examined by Wilcoxon nonparametric tests. To examine the factors affecting weight changes, participants were divided into weight loss or weight gain groups based on their weight change after the intervention. A paired *t*-test was performed to examine individual changes in body composition and biochemical parameters during the intervention. Nonparametric Wilcoxon tests were performed to examine the changes between the weight loss/gain groups. Sleep duration was calculated by subtracting sleeping time from waking time using the collected time and daily dietary records. All statistical analyses were performed using SAS 9.4 (SAS Institute Inc., Cary, NC, USA), and statistical significance was considered when *p* < 0.05.

## 3. Results

### 3.1. Participant Characteristics

Thirty-four participants were enrolled in the intervention and 33 participants completed the 4-week intervention; one woman dropped out during week 2 due to a time schedule conflict. The basic characteristics of the participants are shown in Table 1. The mean age of the subjects was 22.5 years, and the proportion of men was 24%. The average BMI was significantly higher in men than in women (24.5 vs. 22.1). There were no significant differences in alcohol consumption, smoking status, physical activity, or weight control between men and women. Regarding the eating time window, most participants started between 11 a.m. and 1 p.m. and finished around 7–8 p.m. Of the 33 subjects, 17 began their eating time before 12 p.m. and the remaining subjects started after 12 p.m.

### 3.2. Body Composition Measurements

Table 2 shows the body composition changes of the participants during the 4-week intervention. Significant individual changes in body weight (−1.0 ± 1.2 kg), muscle mass (−0.3 ± 0.8 kg), and waist circumference (−1.3 ± 3.3 cm) were found in week 2. After 4 weeks, we observed significant individual changes in body weight (−1.0 ± 1.4 kg), BMI (−0.4 ± 0.5 kg/m^2^), and percent of body fat (−0.4 ± 1.9%). When stratified by sex, the changes were more prominent in women than in men.

**Figure 2:**
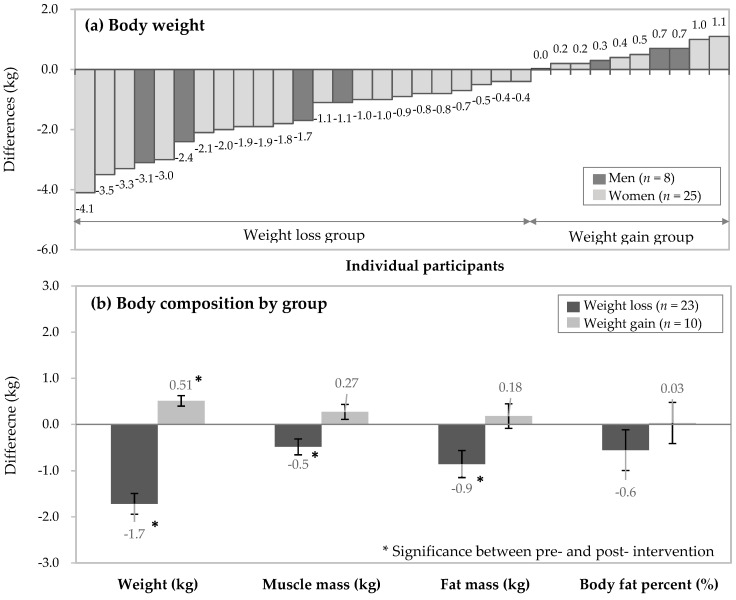
Changes in body composition measures after 4 weeks of time-restricted eating. (**a**) Weight change of each participant; (**b**) average weight change per group.

Individual weight changes are presented in Figure 2a. Weight change after 4 weeks ranged from −4.1 kg to 1.1 kg. Based on weight change after 4 weeks, participants were divided into weight loss (*n* = 23) or weight gain (*n* = 10) groups. The number of men in each group was the same and the dark bars in Figure 2a represent the male participants. The average change in body composition measures per group are presented in Figure 2b. The weight loss group exhibited weight changes from −4.1 to −0.4 kg, showing significant reductions in body weight, muscle mass, and fat mass after 4 weeks. On the other hand, the weight gain groups exhibited weight changes from 0 to 1.1 kg and showed significant increases in body weight, but with no significant increase in muscle or fat mass.

### 3.3. Biochemical Parameters

Changes in cardiometabolic risk factors are presented for the weight loss and weight gain groups in Table 3. Levels of fasting insulin (−3.1 ± 6.3 μU/mL) and HOMA-IR (−0.8 ± 1.6) were significantly lower in the weight loss group after 4 weeks of TRE, but showed no significant changes in the weight gain group after intervention. Regarding blood lipid parameters, LDL-cholesterol levels (13.0 ± 11.7 mg/dL) were significantly higher and HDL-cholesterol levels (−4.3 ± 7.7 mg/dL) were significantly lower in the weight loss group after intervention. However, in the weight gain group, LDL- and HDL-cholesterol levels were higher after 4 weeks of TRE, but this was not significant. Among the cardiometabolic risk factors, fasting insulin, HOMA-IR, and HDL-cholesterol levels were significantly different between the two weight change groups.

### 3.4. Sleep Hours, Sleep Quality, and Psychological Characteristics

Table 4 shows the sleep-related and psychological characteristics of the participants. The average wake-up and sleep times were 9:33 a.m. and 2:08 a.m., respectively, and the average sleep duration was 7.4 h. Chronotype was reported as evening type (54.6%), or morning type (18.2%), and there was no significant difference in chronotype between the weight change groups (*p* = 0.2494). The subjects mostly reported their sleep quality as very good (15.2%) or fairly good (51.5%); most participants fell asleep within 60 min (87.8%), and experienced difficulty in falling asleep within 30 min less than twice a week (72.8%). There was no significant difference in sleep pattern between the weight change groups. Regarding psychological measures, the self-esteem and emotional responsivity scores did not significantly differ between the two groups.

### 3.5. Meal Frequency and Proportions

The meal frequency and meal proportions for the weight loss and gain groups are shown in Table 5. The average number of daily eating occasions during the eating window was 2.8 for all subjects, with no significant difference between the weight change groups. The average meal frequencies during the 28 days of TRE were 3.6 times for breakfast, 24.6 times for lunch, 22.3 times for dinner, and 21.2 times for snacks; there were no significant differences in meal frequencies between the weight change groups.

Meal proportion was evaluated according to nutrient intake per meal. The energy intake per meal (percentage of total energy intake per day) was comprised of 4.5% from breakfast, 39.2% from lunch, 37.6% from dinner, and 18.5% from snacks in all subjects, with no significant differences between the weight change groups. No nutrient intakes were significantly different between the two groups, with the exception of saturated fat intake from dinner. The saturated fat intake from dinner was significantly lower in the weight loss group, who consumed 3.1% of their total energy from saturated fats at dinner, compared to the weight gain group, who received 6.0% of their energy from saturated fats at dinner.

## 4. Discussion

In this four-week dietary intervention of 8 h TRE, we found significant differences in pre- and post-intervention body weight. Subjects were divided into weight loss or weight gain groups based on their weight change after four weeks of TRE, and the weight loss group exhibited a significant reduction in muscle and fat mass, as well as improved glucose control after the intervention; however, these variables did not change significantly in the weight gain group. We demonstrated that TRE is an effective strategy for weight management in healthy young adults, although some subjects (weight gain group, 30%) showed a slight increase in body weight (0 to 1.1 kg) after the intervention. Moreover, participants in the weight loss group experienced body composition changes such as reductions in muscle mass and fat mass.

The effects of TRE on weight change reported by the limited number of existing human studies are inconclusive. Two randomized controlled trials of obese adults reported that the TRE group showed a significant reduction in body weight after 12 weeks compared to the non-TRE or control groups [9,25]. A study of obese adults reported that participants in the 8 h TRE group experienced mild weight loss compared to the control group after 12 weeks [8], and another study of obese adults also reported that 4 h or 6 h TRE caused a mild reduction in weight after 8 weeks. In contrast, studies of adults with prediabetes or metabolic syndrome showed no significant changes in body weight after TRE intervention, although they exhibited improvements in cardiometabolic risk factors such as glucose control, blood pressure, and blood lipids [10,11,26]. Taken together, this suggests that some but not all participants benefit from TRE in terms of weight loss and reducing cardiometabolic risk factors.

Unlike existing studies, in which TRE participants were typically obese or at risk of metabolic disease, our subjects were healthy young adults without obesity. However, our subjects voluntarily participated out of a desire to manage their body weight, as they consumed meals at irregular times due to late-shifted sleeping patterns. Indeed, young adults tend to spend a considerable amount of time on the internet, which may promote late chronotypes. In this study, only half of the subjects categorized themselves as late chronotypes at baseline; however, the time logs of the participants revealed that their average sleeping and waking times were 2 a.m. and 9 a.m., respectively. Our study suggests that TRE can be an effective strategy to manage body weight for individuals with late-shifted sleeping patterns although it is not effective for everyone.

There is currently an ongoing debate regarding the optimal timing of the eating window during TRE interventions. TRE highlights the importance of daily circadian rhythms in regulating the physiological actions of humans. Melatonin secretion and insulin sensitivity are prime examples of processes that are regulated by day/night cycles, in which melatonin secretion increases at night and insulin sensitivity increases during the day [1,27]. Two studies examining early TRE windows reported improved insulin sensitivities; these studies applied an early 6 h TRE window with dinner before 3 p.m. over 5 weeks [26] and an 8 h TRE window from 8 a.m. to 4 p.m. over 2 weeks [28]. By contrast, the results of studies examining late TRE windows were inconsistent. A late TRE study reported no significant differences in body composition changes when TRE was applied in any 4 h window between 4 p.m. and midnight over 8 weeks [29]. However, 4 h (eating between 3 p.m. and 7 p.m.) or 6 h (eating between 1 p.m. and 7 p.m.) TRE windows were effective, leading to significant reductions in body weight and insulin resistance over 8 weeks [30]; the authors speculated that enabling the participants to select a later eating window improved overall compliance. This is in line with our findings; our study revealed positive effects of TRE on weight loss and glucose control, despite the participants having relatively late eating time windows (most subjects started their 8 h window after 11 a.m.). As a result, the compliance of our subjects was very high, as confirmed by the daily diet and sleep time logs recorded via a mobile app. This implies that young adults have late circadian rhythms, and that TRE can be tailored to evening chronotypes.

The meal patterns of the TRE participants were explored by measuring meal frequency and nutrient proportions using the dietary records logged over the 28-day intervention. Regarding meal frequency, participants reported an average of 2.8 daily eating occasions, which was low compared to individuals not undergoing TRE. A study of 14,279 South Korean adults reported that the median number of eating occasions was 5–6 per day [31]. Another study of 19,427 US adults reported average daily eating frequencies of 4.3 in men and 4.2 in women [32]. This indicates that TRE leads to a reduction in meal frequency. However, previous findings regarding the effects of meal frequency on health outcomes have been inconclusive. According to a recent meta-analysis, there is little evidence to suggest that reducing meal frequency is beneficial [33], and that meal frequency is not an independent variable that affects health outcomes. Further studies are necessary to differentiate the effects of meal frequency during TRE.

TRE is an attractive strategy due to its simplicity without calorie counting. In this study, we did not provide participants with any guidance concerning calorie or macronutrient composition. The average energy intake during the intervention was 1529 kcal, and there was no significant difference in the energy intake between the weight loss and weight gain groups. However, when we evaluated nutrient intake by meal, we found that the weight grain group had a significantly higher intake of saturated fat from dinner. According to previous findings, greater energy intake later in the day is significantly associated with lower insulin sensitivity [6,34] and night-time eating (25% or more of total energy consumed after 9 p.m.) is significantly associated with increased prevalence of metabolic syndrome [31]. Further investigations are needed to explore whether dietary quality or meal composition can enhance the positive effects of TRE on weight control and health outcomes.

A major strength of this study is that, to the authors’ knowledge, this is the first study to examine the effects of an 8 h TRE intervention in healthy young adults with late chronotypes. Moreover, not all subjects exhibited weight loss following TRE; the factors affecting weight change in TRE, such as meal patterns, sleep, and psychological factors, were explored between the weight loss/gain groups. We also had high compliance from our participants. Daily diet records and sleep time logs enabled us to track TRE adherence carefully.

Our study also had several limitations. The sample size in this study was small, meaning that findings could not be generalized to all healthy young adults. However, our findings suggest that TRE can be an effective strategy for weight management in healthy young adults. Further studies with larger sample sizes are needed to confirm our findings. Moreover, this was a single-arm study without a control group. However, instead of making comparisons with a control group, we focused on the individual changes between measurements taken before and after the intervention, after adjusting for potential confounding variables. In addition, we did not assess dietary intake before the intervention, which limited our evaluation to the dietary changes that took place during the TRE. To compensate, we obtained 28 days of dietary records that are likely to have represented the usual intakes and meal patterns of the individuals. Lastly, we did not adjust for sex when examining the factors affecting the weight change between the weight loss and gain groups due to the small sample size. However, the weight loss and weight gain group contained the same number of men (four in each group).

## 5. Conclusions

The findings of this four-week dietary intervention of 8 h TRE suggest that TRE is an effective method to manage weight and cardiometabolic risk factors. Although the TRE regimen is not appropriate for everyone, TRE can be an effective lifestyle strategy for managing body weight and cardiometabolic risk factors in young adults with late chronotypes. To strengthen the effect of TRE on weight loss, meal composition may be taken into account. Future studies should examine the effect of TRE in a larger sample over longer time periods to confirm our findings [34].

## Figures and Tables

**Table 1:** The basic characteristics of the time-restricted eating participants.

	Total (*n* = 33)	Men (*n* = 8)	Women (*n* = 25)	*p* ^1^
**Age ^2^** (**years**)	22.5 ± 2.8	24.5 ± 2.7	21.9 ± 2.6	0.0185
**BMI** (**kg/m^2^**)	22.7 ± 2.7	24.5 ± 0.9	22.1 ± 2.9	0.0140
**Alcohol consumption ^3^**				0.0748
Yes	25(75.8)	8(100.0)	17(68.0)	
No	8(24.2)	0(0.0)	8(32.0)	
**Smoking ^4^**				0.0920
None	30(90.9)	6(75.0)	24(96.0)	
Former smoker	1(3.0)	1(12.5)	0(0.0)	
Current smoker	2(6.1)	1(12.5)	1(4.0)	
**Physical activity ^5^**				0.2841
Yes	15(45.5)	5(62.5)	10(40.0)	
No	18(54.6)	3(37.5)	15(60.0)	
**Eating time window**				0.3826
Start before 12:00	17(51.5)	3(37.5)	14(56.0)	
Start after 12:00	16(48.5)	5(62.5)	11(44.0)	

^1^ *p*-values are from the Wilcoxon analysis. ^2^ All values are presented as mean ± SD or *n* (%). ^3^ Alcohol consumption: “Yes,” more than once a month over the past year. ^4^ Smoking: “Current smoker”, more than five packs (100 pieces) in total and smoking now. ^5^ Physical activity: “Yes,” performed vigorous intensity activity for at least 75 min, moderate-intensity activity for at least 150 min, or a combination of moderate and vigorous intensity activities at least 150 min per week.

**Table 2:** Changes of body composition measures after 4 weeks of time-restricted eating.

	Baseline	Week 2	Week 4	Change (2 Weeks)		Change (4 Weeks)	
				Mean ± SD	*p*-Value ^1^	Mean ± SD	*p*-Value ^1^
**Body weight** (**kg**)							
Total (*n* = 33)	62.0 ± 12.1	61.0 ± 11.8	61.0 ± 11.7	−1.0 ± 1.2	<0.0001 *	−1.0 ± 1.4	0.0001 *
Men (*n* = 8)	74.8 ± 5.0	74.1 ± 4.9	73.9 ± 4.8	−0.7 ± 1.0	0.0856	−0.9 ± 1.4	0.1168
Women (*n* = 25)	57.9 ± 10.8	56.8 ± 10.3	56.8 ± 10.1	−1.1 ± 1.2	0.0001 *	−1.1 ± 1.4	0.0007 *
**BMI** (**kg/m^2^**)							
Total (*n* = 33)	22.7 ± 2.7	22.3 ± 2.7	22.3 ± 2.6	−0.4 ± 0.4	<0.0001 *	−0.4 ± 0.5	0.0001 *
Men (*n* = 8)	24.5 ± 0.9	24.3 ± 1.0	24.3 ± 0.9	−0.2 ± 0.3	0.0888	−0.3 ± 0.4	0.1162
Women (*n* = 25)	22.1 ± 2.9	21.7 ± 2.7	21.7 ± 2.7	−0.4 ± 0.4	<0.0001 *	−0.4 ± 0.5	0.0007 *
**Muscle mass** (**kg**)							
Total (*n* = 33)	24.0 ± 6.4	23.7 ± 6.5	23.8 ± 6.4	−0.3 ± 0.8	0.0204 *	−0.3 ± 0.8	0.0750
Men (*n* = 8)	33.6 ± 3.5	33.6 ± 3.5	33.5 ± 3.8	0.0 ± 0.5	0.8490	−0.1 ± 0.6	0.3567
Women (*n* = 25)	21.0 ± 3.4	20.5 ± 3.1	20.7 ± 3.0	−0.4 ± 0.8	0.0109 *	−0.3 ± 0.9	0.0726
**Fat mass** (**kg**)							
Total (*n* = 33)	18.1 ± 5.8	17.7 ± 6.0	17.5 ± 5.8	−0.4 ± 1.1	0.0593	−0.5 ± 1.3	0.0763
Men (*n* = 8)	15.5 ± 2.6	15.0 ± 2.2	14.9 ± 2.6	−0.5 ± 1.4	0.3444	−0.6 ± 1.0	0.8218
Women (*n* = 25)	18.9 ± 6.3	18.6 ± 6.6	18.4 ± 6.3	−0.3 ± 1.0	0.1144	−0.5 ± 1.4	0.0811
**Body fat percent** (**%**)							
Total (*n* = 33)	29.3 ± 7.2	29.1 ± 7.9	28.9 ± 7.8	−0.4 ± 1.6	0.6225	−0.4 ± 1.9	0.0257 *
Men (*n* = 8)	20.8 ± 3.7	20.3 ± 3.4	20.3 ± 4.1	−0.5 ± 1.7	0.4432	−0.5 ± 1.1	0.1344
Women (*n* = 25)	32.0 ± 5.8	32.0 ± 6.7	31.7 ± 6.6	0.0 ± 1.7	0.9147	−0.3 ± 2.1	0.4324
**Waist circumference** (**cm**)							
Total (*n* = 33)	77.0 ± 7.8	75.7 ± 7.9	75.9 ± 7.6	−1.3 ± 3.3	0.0373 *	−1.1 ± 3.5	0.2650
Men (*n* = 8)	84.5 ± 3.6	82.8 ± 4.7	83.3 ± 4.0	−1.7 ± 2.6	0.1000	−1.2 ± 3.4	0.2481
Women (*n* = 25)	74.6 ± 7.3	73.5 ± 7.4	73.5 ± 7.0	−1.1 ± 3.6	0.1326	−1.1 ± 3.6	0.1396

^1^*p*-values were calculated using paired *t*-tests (* *p* < 0.05).

**Table 3:** Changes in biochemical parameters after 4 weeks of time-restricted eating.

	Baseline	Week 4	Absolute Change		Value between Groups	
			Mean ± SD	*p*-Value ^1^	Mean ± SD	*p*-Value ^2^
**Fasting insulin** (**μU/mL**)						
Total (*n* = 33)	10.5 ± 8.0	9.7 ± 9.4	−0.8 ± 9.0	0.6098		
Weight loss (*n* = 23)	11.9 ± 8.8	8.7 ± 5.2	−3.1 ± 6.3	0.0266 *	−7.7 ± 8.4	0.0312 *
Weight gain (*n* = 10)	7.9 ± 4.7	11.8 ± 15.6	4.6 ± 12.0	0.2601		
**Fasting glucose** (**mg/dL**)						
Total (*n* = 33)	95.8 ± 6.6	94.1 ± 6.7	−1.7 ± 5.5	0.0875		
Weight loss (*n* = 23)	95.7 ± 7.1	93.7 ± 6.3	−2.0 ± 5.8	0.1221	0.9 ± 5.6	0.8289
Weight gain (*n* = 10)	96.1 ± 5.8	95.0 ± 7.9	−1.1 ± 5.0	0.5041		
**HOMA-IR ^3^**						
Total (*n* = 33)	2.5 ± 2.1	2.3 ± 2.5	−0.2 ± 2.3	0.6145		
Weight loss (*n* = 23)	2.9 ± 2.3	2.1 ± 1.3	−0.8 ± 1.6	0.0252 *	2.0 ± 2.2	0.0269 *
Weight gain (*n* = 10)	1.8 ± 1.2	2.9 ± 4.1	1.2 ± 3.1	0.2711		
**Total cholesterol** (**mg/dL**)						
Total (*n* = 33)	191.2 ± 32.2	193.9 ± 33.6	2.7 ± 14.3	0.2873		
Weight loss (*n* = 23)	188.2 ± 21.3	192.3 ± 25.4	4.1 ± 14.8	0.1985	−4.6 ± 14.4	0.3772
Weight gain (*n* = 10)	198.1 ± 50.1	197.6 ± 49.2	−0.5 ± 13.4	0.9083		
**Triglyceride** (**mg/dL**)						
Total (*n* = 33)	99.7 ± 50.2	94.1 ± 51.0	−5.6 ± 56.3	0.5712		
Weight loss (*n* = 23)	100.3 ± 52.9	90.2 ± 34.2	−10.2 ± 49.9	0.3385	15.1 ± 56.7	0.6952
Weight gain (*n* = 10)	98.3 ± 45.9	103.2 ± 79.2	4.9 ± 70.8	0.8316		
**LDL-cholesterol** (**mg/dL**)						
Total (*n* = 33)	109.5 ± 29.9	119.5 ± 28.6	10.1 ± 13.4	0.0001 *		
Weight loss (*n* = 23)	106.7 ± 23.0	119.7 ± 22.7	13.0 ± 11.7	<0.0001 *	−9.5 ± 12.9	0.0569
Weight gain (*n* = 10)	115.7 ± 42.8	119.2 ± 40.7	3.5 ± 15.3	0.4870		
**HDL-cholesterol** (**mg/dL**)						
Total (*n* = 33)	65.5 ± 17.0	63.2 ± 15.7	−2.4 ± 8.3	0.1070		
Weight loss (*n* = 23)	65.1 ± 18.5	60.7 ± 14.3	−4.3 ± 7.7	0.0130 *	6.4 ± 7.8	0.0840
Weight gain (*n* = 10)	66.6 ± 13.5	68.7 ± 18.0	2.1 ± 8.2	0.4373		

^1^ *p*-values are from the paired *t*-test, and ^2^ *p*-values are from the Wilcoxon analysis (* *p* < 0.05). ^3^ HOMA-IR stands for Homeostatic Model Assessment of Insulin Resistance.

**Table 4:** Sleep and psychological characteristics of participants during 4 weeks of time-restricted eating.

	Total (*n* = 33)	Weight Loss (*n* = 23)	Weight Gain (*n* = 10)	*p* ^1^
**Sleep hours**				
Wake-up time ^2^ (hh:mm)	9:33 ± 2:03	9:32 ± 2:04	9:35 ± 2:01	0.2350
Bedtime (hh:mm)	2:08 ± 1:48	2:14 ± 1:55	1:52 ± 1:30	0.1253
Sleep duration (h)	7.4 ± 0.8	7.3 ± 0.8	7.7 ± 0.7	0.2172
**Chronotype** **(*n*, (%))**				0.2494
Morning type	6(18.2)	5(21.7)	1(10.0)	
Evening type	18(54.6)	13(56.5)	5(50.0)	
No matter	9(27.3)	5(21.7)	4(40.0)	
**Sleep quality (*n*, (%))**				0.2186
Very good	5(15.2)	4(17.4)	1(10.0)	
Fairly good	17(51.5)	13(56.5)	4(40.0)	
Fairly bad	11(33.3)	6(26.1)	5(50.0)	
**Time taken to fall asleep** **(*n*, (%))**				0.8202
≤15 min	7(21.2)	5(21.7)	2(20.0)	
16–30 min	8(24.2)	5(21.7)	3(30.0)	
31–60 min	14(42.4)	10(43.5)	4(40.0)	
≥60 min	4(12.1)	3(13.0)	1(10.0)	
**Cannot get to sleep within 30 min** **(*n*, (%))**				0.7297
None	5(15.2)	5(21.7)	0(0.0)	
<Once a week	10(30.3)	4(17.4)	6(60.0)	
Once or twice a week	9(27.3)	7(30.4)	2(20.0)	
Three or more times a week	9(27.3)	7(30.4)	2(20.0)	
**Psychological measures**				
Self-esteem stability	27.0 ± 7.4	26.0 ± 8.1	29.3 ± 5.3	0.3564
Emotional responsivity	21.0 ± 13.5	22.5 ± 13.4	17.5 ± 13.8	0.2720
Emotional sensitivity	9.9 ± 6.7	11.1 ± 6.9	7.0 ± 5.6	0.0676
Emotional intensity	7.2 ± 5.3	7.1 ± 5.0	7.2 ± 6.2	0.7979
Emotional persistence	3.9 ± 2.8	4.2 ± 2.9	3.3 ± 2.7	0.4169

^1^ *p*-values were calculated using Wilcoxon tests. ^2^ Continuous variables are presented as mean ± SD or *n* (%).

**Table 5:** Meal frequency and meal proportions in 4 weeks of time-restricted eating.

	Total (*n* = 33)	Weight Loss (*n* = 23)	Weight Gain (*n* = 10)	*p* ^1^
**Daily eating occasion ^2^**	2.8 ± 0.5	2.8 ± 0.5	2.8 ± 0.6	0.9531
**Meal frequency**				
Breakfast (No. over 28 days)	3.6 ± 8.2	3.9 ± 8.7	2.9 ± 7.5	0.7500
Lunch (No. over 28 days)	24.6 ± 3.8	24.8 ± 3.8	24.2 ± 3.9	0.6610
Dinner (No. over 28 days)	22.3 ± 8.2	21.0 ± 9.4	25.2 ± 3.3	0.3117
Snack (No. over 28 days)	21.2 ± 7.7	21.7 ± 7.4	19.8 ± 8.6	0.7825
**Meal proportion** (**nutrient intake per meal**)				
**Breakfast**				
Energy (kcal/day)	72.8 ± 180.2	71.9 ± 163.9	75.0 ± 222.9	0.6659
Energy (% of daily energy intake)	4.5 ± 10.9	4.7 ± 10.9	4.1 ± 11.5	0.6990
Carbohydrate (%)	2.8 ± 8.3	3.7 ± 9.7	0.8 ± 2.6	0.5721
Sugar (%)	0.3 ± 0.9	0.4 ± 1.1	0.1 ± 0.2	0.8089
Protein (%)	0.7 ± 1.9	0.6 ± 1.7	0.7 ± 2.3	0.7064
Fat (%)	1.5 ± 4.3	1.3 ± 3.4	1.9 ± 6.0	0.7064
Saturated fat (%)	0.5 ± 1.5	0.5 ± 1.3	0.6 ± 1.8	0.6603
**Lunch**				
Energy (kcal/day)	588.2 ± 177.2	583.9 ± 171.5	598.1 ± 199.1	0.9844
Energy (% of daily energy intake)	39.2 ± 9.4	40.4 ± 9.9	36.6 ± 7.7	0.3372
Carbohydrate (%)	19.9 ± 15.9	19.6 ± 16.7	20.5 ± 14.5	0.6521
Sugar (%)	3.7 ± 3.9	3.9 ± 4.1	3.3 ± 3.7	0.7687
Protein (%)	7.3 ± 5.5	6.7 ± 5.9	8.7 ± 4.6	0.2812
Fat (%)	11.2 ± 11.4	10.1 ± 12.5	13.5 ± 8.5	0.0883
Saturated fat (%)	3.3 ± 3.8	2.9 ± 4.1	4.0 ± 2.7	0.1039
**Dinner**				
Energy (kcal/day)	592.1 ± 251.5	547.2 ± 266.7	695.3 ± 184.5	0.1040
Energy (% of daily energy intake)	37.6 ± 15.3	35.7 ± 16.5	42.0 ± 11.6	0.3997
Carbohydrate (%)	23.7 ± 20.2	25.0 ± 22.6	20.9 ± 13.7	0.9219
Sugar (%)	5.3 ± 9.1	4.9 ± 10.4	6.0 ± 5.4	0.2634
Protein (%)	6.7 ± 4.4	6.4 ± 4.8	7.4 ± 3.7	0.4923
Fat (%)	12.9 ± 10.4	11.7 ± 11.5	15.7 ± 7.0	0.2022
Saturated fat (%)	4.0 ± 3.3	3.1 ± 3.2	6.0 ± 2.5	0.0241 *
**Snack**				
Energy (kcal/day)	274.0 ± 168.1	277.2 ± 167.5	266.6 ± 178.3	0.7392
Energy (% of daily energy intake)	18.5 ± 11.9	19.1 ± 11.9	17.0 ± 12.3	0.4449
Carbohydrate (%)	7.4 ± 8.8	7.8 ± 9.5	6.3 ± 7.3	0.7380
Sugar (%)	3.1 ± 4.0	3.0 ± 4.3	3.3 ± 3.3	0.6778
Protein (%)	1.4 ± 1.7	1.4 ± 1.8	1.3 ± 1.7	0.6932
Fat (%)	4.6 ± 5.4	5.7 ± 5.8	2.2 ± 3.3	0.3353
Saturated fat (%)	1.8 ± 2.3	2.1 ± 2.5	1.0 ± 1.4	0.6018

^1^ *p*-values were calculated via Wilcoxon analysis. ^2^ Daily eating occasions were counted from 0 to 7 occasions including breakfast, lunch, dinner, and a light snack before breakfast, a morning snack, an afternoon snack, or a late snack.

## Data Availability

The data presented in this study are available on request from the corresponding author. The data are not publicly available due to privacy.
